# Participation and performance trends in ‘Ultraman Hawaii’ from 1983 to 2012

**DOI:** 10.1186/2046-7648-2-25

**Published:** 2013-08-01

**Authors:** Dimirela Meili, Beat Knechtle, Christoph Alexander Rüst, Thomas Rosemann, Romuald Lepers

**Affiliations:** 1 Institute of General Practice and for Health Services Research, University of Zurich, Pestalozzistrasse 24, Zurich 8091, Switzerland; 2 Facharzt FMH für Allgemeinmedizin, Gesundheitszentrum St. Gallen, Vadianstrasse 26, St. Gallen 9001, Switzerland; 3 INSERM U1093, Faculty of Sport Sciences, University of Burgundy, UFR STAPS, BP 27877, Dijon Cedex 21078, France

**Keywords:** Ultra-endurance, Swimming, Cycling, Running, Sex difference, Age, Triathlete

## Abstract

**Background:**

Participation and performance trends have been investigated in a single stage Ironman triathlon such as the ‘Ironman Hawaii,’ but not for a multi-stage ultra-triathlon such as the ‘Ultraman Hawaii’ covering a total distance of 515 km. The aims of this study were to analyze (1) changes in participation and performance, (2) sex-related differences in overall and split time performances, and (3) the age of peak performance in Ultraman Hawaii.

**Methods:**

Age and race times including split times for 98 women and 570 men who successfully finished Ultraman Hawaii (day 1 with 10-km swimming and 145-km cycling, day 2 with 276-km cycling, and day 3 with 84-km running) between 1983 and 2012 were analyzed. Changes in variables over time of annual winners and annual top three women and men were investigated using simple linear regression analyses.

**Results:**

The number of female finishers increased (*r*^2^ = 0.26, *p* < 0.01), while the number of male finishers remained stable (*r*^2^ = 0.03, *p* > 0.05). Overall race times decreased for both female (*r*^2^ = 0.28, *p* < 0.01) and male (r^2^ = 0.14, *p* < 0.05) winners and for both the annual top three women (*r*^2^ = 0.36, *p* < 0.01) and men (r^2^ = 0.14, *p* = 0.02). The sex difference in performance decreased over time from 24.3% to 11.5% (*r*^2^ = 0.39, *p* < 0.01). For the split disciplines, the time performance in cycling on day 1 (*r*^2^ = 0.20, *p* < 0.01) and day 2 decreased significantly for men (*r*^2^ = 0.41, *p* < 0.01) but for women only on day 2 (*r*^2^ = 0.45, *p* < 0.01). Split times showed no changes in swimming and running. The age of the annual winners increased from 28 to 47 years for men (*r*^2^ = 0.35, *p* < 0.01) while it remained stable at 32 ± 6 years for women (*r*^2^ < 0.01, *p* > 0.05). The age of the annual top three finishers increased from 33 ± 6 years to 48 ± 3 years for men (*p* < 0.01) and from 29 ± 7 years to 49 ± 2 years for women (*p* < 0.01).

**Conclusions:**

Both the annual top three women and men improved performance in Ultraman Hawaii during the 1983–2012 period although the age of the annual top three women and men increased. The sex-related difference in performance decreased over time to reach approximately 12% similar to the reports of other endurance and ultra-endurance events. Further investigations are required to better understand the limiting factors of the multi-activities ultra-endurance events taking place over several days.

## Background

Triathlon is an endurance athletic competition consisting of sequential and continuous swimming, cycling, and running events. Triathlon races commonly range from short-distance races such as the ‘Olympic distance’ of 1.5-km swimming, 40-km cycling, and 10-km running to long-distance races such as the ‘Ironman distance’ of 3.8-km swimming, 180-km cycling, and 42.2-km running [1]. Ultra-triathlons are of longer distances than the Ironman, such as the Double Iron ultra-triathlon distance of 7.6-km swimming, 360-km cycling, and 84.4-km running, the Triple Iron ultra-triathlon distance of 11.4-km swimming, 540-km cycling, and 126.6-km running, and the Deca Iron ultra-triathlon distance of 38-km swimming, 1,800-km cycling, and 420-km running [2].

During the past decade, participation increased in ultra-endurance races such as ultra-marathons [3,4] and ultra-triathlons [2,5]. Hoffman and Wegelin [3] reported an increase in starters between 1974 and 1986 in the ‘Western States 100-Mile Endurance Run,’ with stable entries thereafter. Among the overall increase in participation, female participation increased from 10–12% in the late 1980s to 20–22% in 2001, where it remained to the present. Similar findings have been observed for ‘Ironman Hawaii’ [6]. Each year, more than 1,700 triathletes with approximately 27% women were participating in Ironman Hawaii, the Ironman triathlon World Championship held each year [6]. In triathlons longer than the Ironman distance, the percentage of women is lower. The number of female starters in Double Iron ultra-triathlon slightly increased in the last 10 years and counted for approximately 10%. For the Triple Iron ultra-triathlon distance and the Deca Iron ultra-triathlon distance, women starters were, from the beginning of the events, at 8% and 10%, respectively [2].

It has been suggested that women might outrun men with increasing length of an endurance performance [7-9]. Female ultra-marathoners improved performance over time [3]; however, a sex-related difference in performance still remained [10]. Indeed, men were faster than women, and longer distances were associated with greater sex-related differences in performance [8]. For example, men were approximately 19% faster than women in both a Double and Triple Iron ultra-triathlon, and approximately 30% faster in a Deca Iron ultra-triathlon [2]. Furthermore, the sex-related difference in overall race time for winners in a Triple Iron ultra-triathlon increased from 10% in 1992 to 42% in 2011 [11]. In triathlon, the sex-related difference in performance varied among the three locomotion modes. For example, the overall race times of the top ten men in Ironman Hawaii averaged 12.6% faster than those of the top ten women, but average swimming, cycling, and running times differed by 9.8%, 12.7%, and 13.3%, respectively [6]. Race distance might differentially affect the sex gap in the three locomotion modes [12].

Age has been reported as a limiting variable in endurance performance. Performance in endurance competitions such as marathon running decreased with increasing age [13]. Previous studies reported that peak endurance performance was maintained until the age of 30–35 years, followed by a moderate decline until the age of 50–60 years and then a progressively steeper decline after the age of 70–75 years, independent of the discipline and the length of the event [14,15]. The age of peak triathlon performance seemed to depend on the race distance. For example, in ‘Ironman Switzerland’ as a qualifier for Ironman Hawaii, the age of the top ten finishers was 25–39 years for women and 18–39 years for men [16]. Furthermore, in Ironman Hawaii, the age of the overall top ten finishers increased between 1983 and 2012 from 26 ± 5 years to 35 ± 5 years for women and from 27 ± 2 years to 34 ± 3 years for men [17]. In Triple Iron and Deca Iron ultra-triathlons, the age of peak performance has been found between 25 years and 44 years for men [18]. In addition, the mean age of male finishers appeared significantly higher for Deca Iron ultra-triathletes with 41.3 ± 3.1 years than for Triple Iron ultra-triathletes with 38.5 ± 3.3 years [18].

A few studies investigated participation and performance trends in ultra-endurance events held over different stages during several days, i.e., with rest between stages [19-21]. Apart of multi-stage races consisting in single disciplines such as the ‘Tour de France’ or the ‘Vuelta a Espana’ [22,23], a new kind of multi-stage ultra-endurance race was launched in 2006 for ultra-distance triathlon, the ‘World Challenge Deca Iron Triathlon’ [19,20] where athletes have to perform the daily distance of one Ironman triathlon of 3.8-km swimming, 180-km cycling, and 42.2-km running for ten consecutive days [21]. It was concluded that performance in a Deca Iron ultra-triathlon decreased throughout the competition, with the fastest race on day 1 and the slowest on day 10 [21].

In contrast to conventional ultra-triathlons held without stops between the three disciplines [2], the ‘Ultraman’ triathlon taking place in Hawaii as ‘Ultraman Hawaii’ is divided into three stages and is held over three days with a total distance close to a Double Iron ultra-triathlon distance [2] with 10-km swimming and 145-km cycling on day 1, 276-km cycling on day 2, and 84-km running on day 3. The first Ultraman Hawaii was held in 1983 [24]. The ‘Ultraman Triathlon’ is unique in the field and provides therefore an intriguing opportunity to examine the sex-related effects on performance in a multi-stage ultra-endurance event. Previous studies analyzed participation and performance trends in the Ironman distance triathlon [5,6], but no studies examined these trends in multi-stage ultra-triathlons such as Ultraman Hawaii. Since both Ironman Hawaii and Ultraman Hawaii are of high interest for ultra-endurance athletes, participation and performance trends in Ultraman Hawaii need to be investigated and compared to existing findings for Ironman Hawaii.

Therefore, the aims of the present study were to examine (1) the participation and performance trends, (2) the sex-related difference in performance, and (3) the age of peak performance in Ultraman Hawaii during its 20 years of history. Race results from 1983–2012 were analyzed to test the hypotheses whether (1) the participation in women would increase over time, (2) the performance of both sexes would improve over time, (3) the sex-related differences in overall performance would decrease over time, and (4) the age of peak performance would increase over time.

## Methods

The present study included all athletes who successfully finished Ultraman Hawaii between 1983 and 2012, excluding the years 1987 and 1991, where no races were held. Sex, age, and overall race time were obtained from the event website [24]. Data were available for a total of 671 athletes including 98 women (14.6%) and 573 men (85.4%). No information was given for the age for three men who started in 1998; therefore, they were excluded from the analysis. Ultimately, complete data with age and overall race times including split times for 98 women and 570 men who successfully finished Ultraman Hawaii between 1983 and 2012 were included in the analysis. All procedures used in the study met the ethical standards of the Swiss Academy of Medical Sciences [25] and were approved by the Institutional Review Board of Kanton St. Gallen, Switzerland (decision letter of 1 June 2010), with a waiver of the requirement for informed consent of the participants given the fact that the study involved the analysis of publicly available data.

### The race

The annual edition of Ultraman Hawaii is limited to 40 athletes on an invitation-only basis and attracts participants from around the world. Participants must complete a 10-km open ocean swim, a 421-km cross-country non-drafting cycling, and an 84-km ultra-marathon. Each athlete has to be accompanied over the entire course by a support team of at least two persons. The events start around sunrise on each day. The swimming and first cycling events are held on day 1. Average water temperature for the open ocean swim is 21°C, and wetsuits are allowed. Following the swimming event, the participants have to cycle 145 km, with an elevation gain of approximately 2,500 m. On day 2, the participants cycle 276 km, with an elevation gain of approximately 2,600 m. On day 3, the athletes need to complete an 84-km ultra-marathon with an elevation gain of approximately 168.5 m. Each stage must be completed within 12 h.

### Data analysis

Annual female and male winners (i.e., athletes with the fastest overall race time) and the annual overall top three women and men were analyzed to determine changes in the fastest overall race times and in the age of the fastest finishers during the 1983–2012 period. Participation and performance trends in the three split disciplines were analyzed only between 2001 and 2012 due to insufficient data in earlier years. The sex-related difference in performance was calculated using the equation ([race time in women] – [race time in men]) / [race time in men] × 100. The sex-related differences were transformed into absolute values prior to analysis. To calculate the mean sex-related difference in performance between the annual top three women and women, the differences for each pair (e.g., first man and first woman, second man and second woman, third man and third woman) were calculated separately and averaged. The age of the athletes was calculated by year of race – year of birth.

### Statistical analysis

To optimize the reliability of data analysis, each set of data was tested for homogeneity of variances prior to statistical analysis, using Levene’s test in cases of two groups and Bartlett’s test in cases of more than two groups. Linear regression was used to determine the significance of changes in a variable (speed, age) across years. The variables between two groups were compared using unpaired *t* test. Statistical analyses were performed using IBM SPSS Statistics (Version 19, IBM SPSS, Chicago, IL, USA) and GraphPad Prism (Version 5, GraphPad Software, La Jolla, CA, USA). Statistical significance was accepted at *p* < 0.05 (two-sided for *t* tests). Data in the text are given as mean ± standard deviation (SD).

## Results

### Participation trends

**Figure 1:**
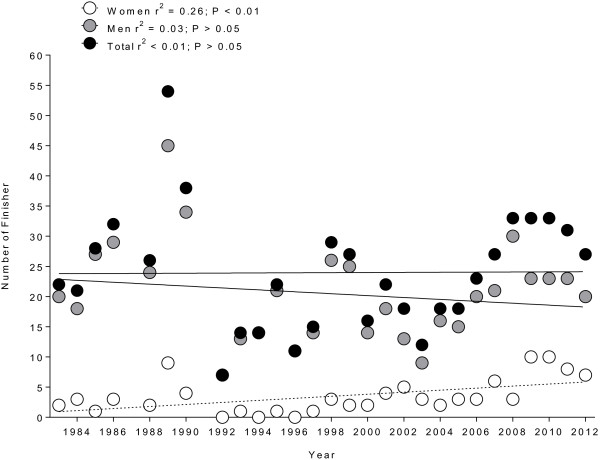
**Changes in the annual number of male, female, and overall finishes in Ultraman Hawaii.** From 1983 to 2012.

**Figure 2:**
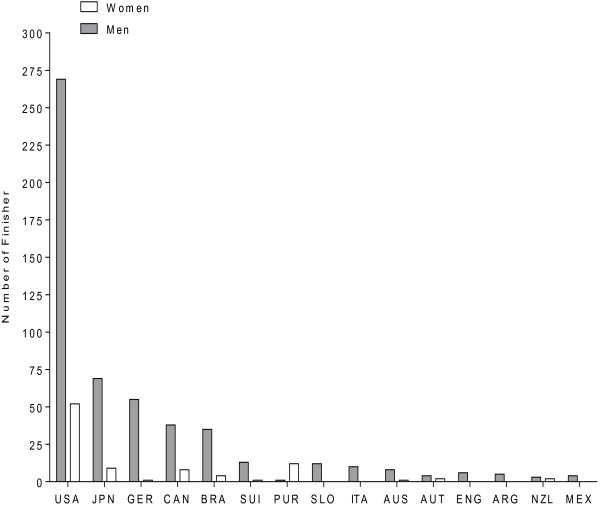
**Number of male and female finishes per country in Ultraman Hawaii between 1983 and 2012.** The frame indicates the 15 countries with the highest number of male and female finishes. Athletes from these 15 countries accounted for 92.7% of all finishers.

Between 1983 and 2012, the annual number of male finishers in Ultraman Hawaii remained unchanged (*r*^2^ = 0.03, *p* > 0.05), while the number of female finishers increased over time (*r*^2^ = 0.26, *p* < 0.01) (Figure 1). The number of female finishers increased from three women (14% of the total field) in 1984 to seven women (26% of the total field) in 2012. The largest number of female finishers (26.5%) was in the age group of 35–39 years, while the largest number of male finishers (24%) was in the age group of 40–44 years. Athletes from 15 countries accounted for 93% of all finishes. The largest number of male finishers was achieved by participants from USA (48%), followed by participants from Japan, Germany, Canada, and Brazil (Figure 2). Most of the female finishers (54.7%) originated from the USA, followed by athletes from Puerto Rico, Japan, Canada, and Brazil.

### Performance trends and sex difference in performance

**Figure 3:**
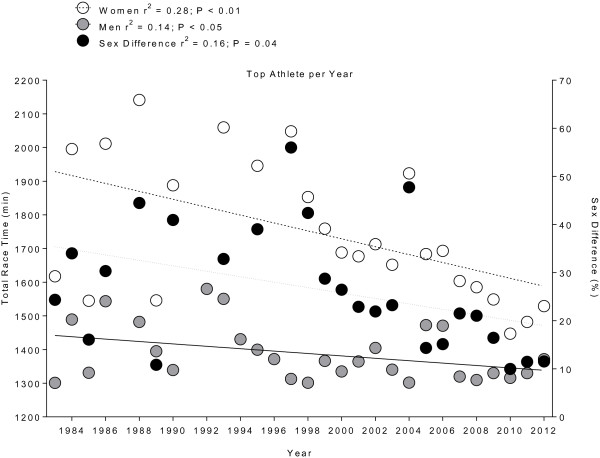
Changes in overall race time with sex difference of the annual top women and men from 1983 to 2012

**Figure 4:**
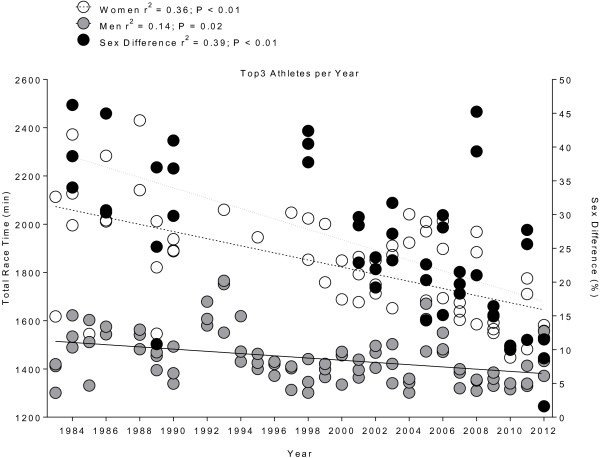
**Changes in overall race time with sex difference of the annual top three women and men from 1983 to 2012.** Values are means ± SD.

**Figure 5:**
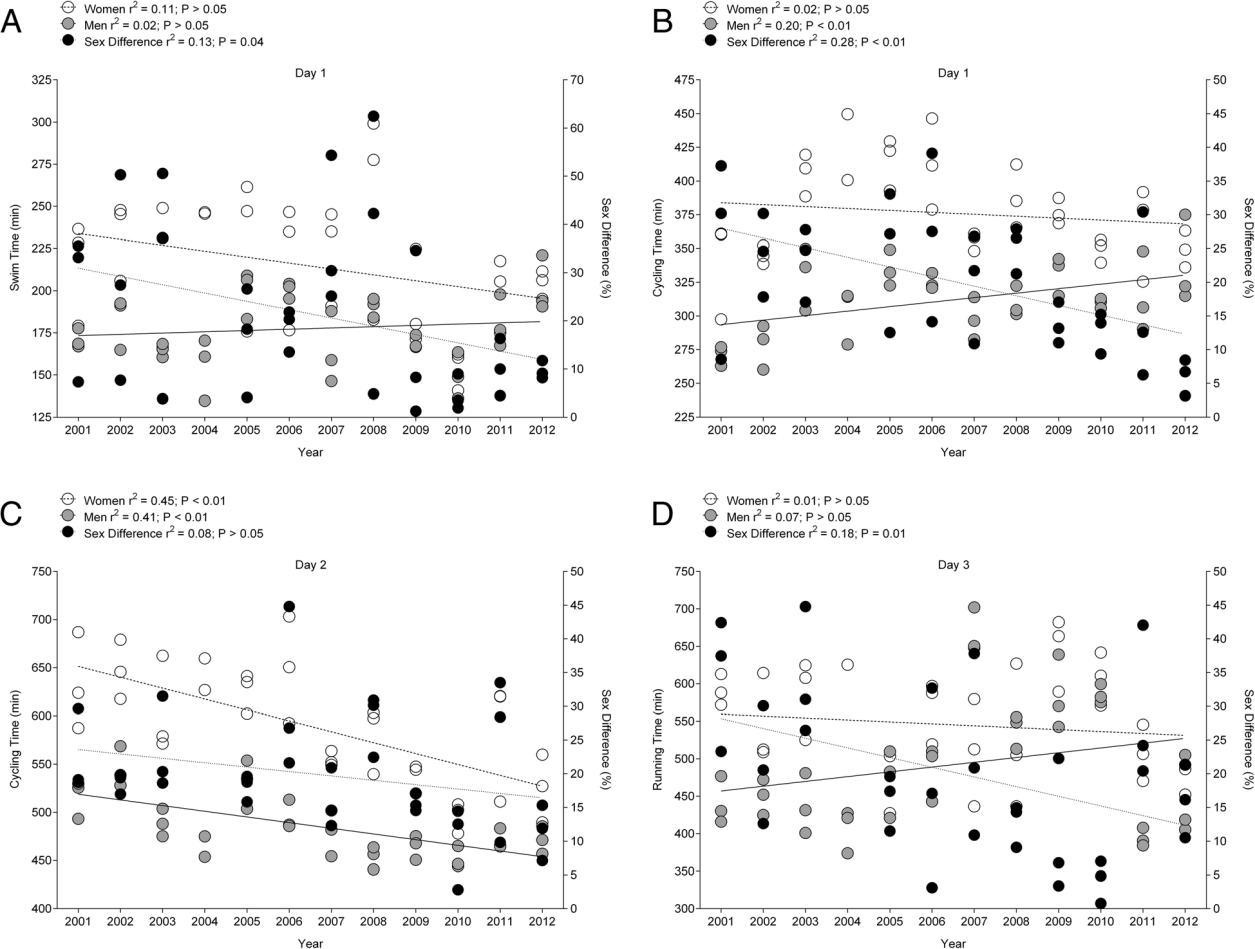
**Changes in performance during the three days.** Panel **A**: Swim time on Day 1, Panel **B**: Cycling time on Day 1, Panel **C**: Cycling time on Day 2, Panel **D**: Running time on Day 3 and corresponding sex difference of the annual top three women and men from 2001 to 2012. Values are means ± SD.

For the annual winners, the overall race time decreased across years for both women (*r*^2^ = 0.28, *p* < 0.01) and men (*r*^2^ = 0.14, *p* < 0.05) (Figure 3). The sex-related difference in overall race time decreased from 24.3% in 1983 to 11.5% in 2012 (*r*^2^ = 0.16, *p* = 0.04). When the top three finishers of each sex were considered, performance improved for both women (*r*^2^ = 0.36, *p* < 0.01) and men (*r*^2^ = 0.14, *p* = 0.02) (Figure 4). The sex-related difference in race time decreased over time (*r*^2^ = 0.39, *p* < 0.01). Figure 5 presents the change in performance for swimming on day 1 (Figure 5A), cycling on day 1 (Figure 5B), cycling on day 2 (Figure 5C), and running on day 3 (Figure 5D). During the 2001–2012 period, the swim time on day 1 (Figure 5A) remained unchanged for the annual top three women (*r*^2^ = 0.11, *p* > 0.05) and men (*r*^2^ = 0.02, *p* > 0.05). However, the sex difference in swimming performance decreased (*r*^2^ = 0.13, *p* = 0.04). The cycling time on day 1 (Figure 5B) showed no change for women (*r*^2^ = 0.02, *p* > 0.05) but decreased in men (*r*^2^ = 0.20, *p* < 0.01). The sex difference in cycling performance decreased significantly by 1.4% *per annum* (*r*^2^ = 0.28, *p* < 0.01). In contrast, the cycling time on day 2 (Figure 5C) decreased over time from 632 ± 50 min to 525 ± 35 min for women (*r*^2^ = 0.45, *p* < 0.01) and from 516 ± 20 min to 471 ± 14 min for men (*r*^2^ = 0.41, *p* < 0.01), while the sex differences remained stable (*r*^2^ = 0.08, *p* > 0.05). On day 2, women improved cycling performance by 11 min and men by 6 min *per annum*. For the marathon on day 3 (Figure 5D), sex difference decreased (*r*^2^ = 0.18, *p* = 0.01), but running time showed no change for both women (*r*^2^ = 0.01, *p* > 0.05) and men (*r*^2^ = 0.07, *p* > 0.05).

### Age of peak performance

**Figure 6:**
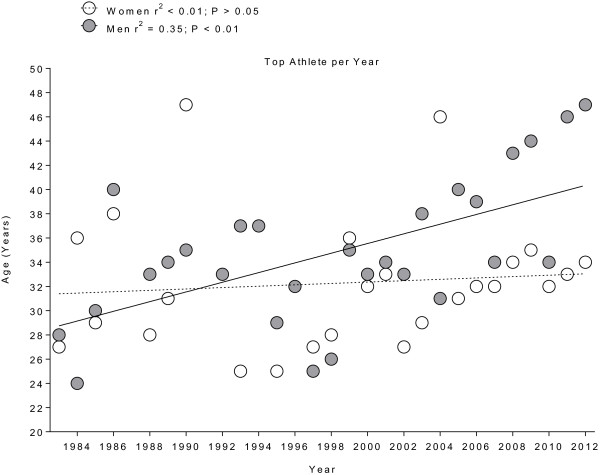
Changes in the age of the annual top women and men from 1983 to 2012.

**Figure 7:**
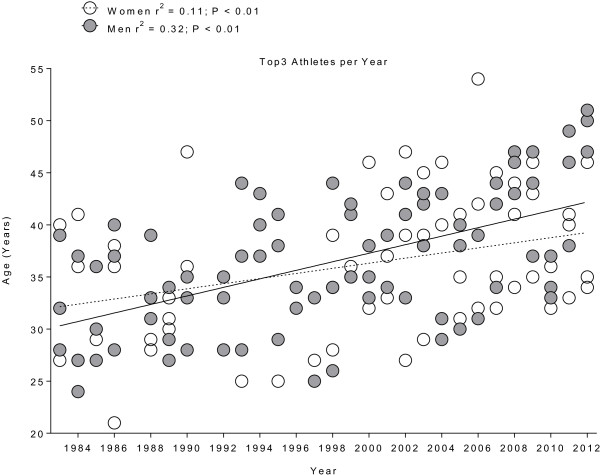
**Changes in the age of the annual top three women and men from 1983 to 2012.** Values are means ± SD.

The mean age of overall finishers was lower for women with 32 ± 5 years compared to men with 35 ± 6 years (*p* < 0.001). The age of the annual winners increased from 28 to 47 years (*r*^2^ = 0.35, *p* < 0.01) for men, while it remained stable at 32 ± 6 years (*r*^2^ < 0.01, *p* > 0.05) for women (Figure 6). The age of the annual top three finishers increased from 33 ± 6 years to 48 ± 3 years for men (*r*^2^ = 0.32, *p* < 0.01) and from 29 ± 7 years to 49 ± 2 years for women (*r*^2^ = 0.11, *p* < 0.01) (Figure 7).

## Discussion

The aims of the present study were to examine (1) trends in participation and performance, (2) the sex-related differences in performance, and (3) the age of peak performance in Ultraman Hawaii between 1983 and 2012. The main findings were (1) the number of female finishers increased over time, (2) overall race times of the annual winners and the annual three fastest finishers decreased over time for both women and men, (3) the sex-related difference in performance decreased over time, and (4) the age of the annual three fastest women and men increased over time.

### Participation trends

The results of the present study supported the hypothesis that the number of female finishers in Ultraman Hawaii increased during 1983–2012, while the number of male finishers remained relatively constant. The number of female finishers in ultra-endurance events generally increased over time, as is evident in Ultraman Hawaii with two female participants in 1983 and seven in 2012, as well as in Ironman Hawaii with 20 female participants in 1981 and more than 450 in 2007, respectively [6]. The reasons for the increase in female participation are not clear, but sociological phenomena such as an increased focus on physical fitness and on the positive influence of healthy living on longevity might be potential explanations [26]. For female ultra-marathoners, the strongest sources of motivation were health and personal achievement [27]. Men, on the other side, rather search for competition and try to reach high rankings. Lombardo suggested that sport began as a way for men to develop the skills needed in primitive hunting and warfare, then developed to act primarily as a competition where athletes display and male spectators evaluate the qualities of potential allies and rivals [28]. Successful ultra-endurance performance is characterized by the ability to sustain a higher absolute speed for a given distance than other competitors [29]. Hunter and Stevens [30] reported for marathoners that the greater sex difference in velocity occurring with age and lower performance was primarily explained by the lower number of female finishers compared to male finishers. These data provided evidence that a lower participation among female competitors could amplify the sex difference in running velocity above that due to physiological sex difference alone. In Ultraman Hawaii, the largest number of finishers originated from the USA, probably because long triathlons offered worldwide provide athletes from Europe and other parts of the world the opportunity to participate without having to travel such long distances [31].

### Changes in overall performance

Overall race time for both sexes decreased significantly during 1983–2012, supporting our second hypothesis. The improvement in performance over time by both sexes in Ultraman Hawaii might be related to an increase in participation in ultra-triathlon events. The increasing number of athletes was probably due to improvements in training, nutrition, and experience-related pacing strategies [6]. During the last century, there has been a continuous decrease in performance times in different sports disciplines such as track and field and swimming [32,33]. Previous experience seemed to play an important role in ultra-endurance performance. Overall race time in a Triple Iron ultra-triathlon was more closely associated with personal best time in previous ultra-triathlons than with anthropometry or training [34]. Race time in a Deca Iron ultra-triathlon was also more closely related to the number of finished Triple Iron ultra-triathlons and personal best time than to anthropometric characteristics [21]. Considering these findings, the number of successfully finished Triple Iron ultra-triathlons and a fast personal best time in Triple Iron ultra-triathlon might reflect pre-race training volume and experience, where athletes with a higher training volume might compete faster in a longer ultra-triathlon than athletes with a lower training volume and less experience.

### Changes in sex-related differences in performance

The sex-related difference in overall performance for both the top three athletes and the annual winners decreased over time. The annual top three women and men improved cycling time. The sex difference in performance decreased for day 1 annually by 1.4%. On day 2, both women and men became faster over time, but the sex difference in performance remained unchanged. Female athletes performed by 11 min faster *per annum*, while male by 6 min *per annum*. One third of the Ultraman Hawaii distance consists of cycling, so this mode of locomotion has a strong influence on overall race time. Cycling technology has improved consistently since 1981 [35], probably contributing to improved performance by both sexes in the cycling portion of the race. Based on the single top finishers and the top three finishers of each sex, men consistently performed better than women in Ultraman Hawaii.

Long-distance events were generally associated with large sex-related differences in performance [4,8,11,36]. Men were physically stronger and had greater aerobic capacity than women [37]. Men also tended to have stronger ego involvement in athletic competitions than women [38]. Female ultra-marathoners were more task-orientated than ego-orientated and set goals other than winning [27]. Deaner [39] and Deaner and Mitchell [40] argued that the ultimate benefit of studying sex differences in enduring competitiveness from an evolutionary perspective is not to support an essentialist position. The payoff, instead, should be improved in the understanding of the various factors that affect long-term achievement motivation. Deaner showed the importance of sex but also highlighted that motivation should be modulated by whether achievement in the domain yields status or resources and whether one perceives that they possess talent in the domain [39]. In particular, men should be relatively more motivated to achieve in context where their talent allows them to compete for status whereas women would be relatively more likely to compete for resources.

Previous studies investigating performance in ultra-triathlons showed that changes in male and female performance varied among the three locomotion modes. In Ironman Hawaii, for example, the sex-related difference remained stable in swimming (+0.1% per decade), increased slightly in cycling (+0.8% per decade), and decreased in running (−2.8% per decade) between 1981 and 2007 [6]. Rüst et al. showed that for Ironman Hawaii from 1983 to 2012, the sex differences decreased over years for overall race time and running but not for swimming and cycling [17]. Sex-related differences in running were predicted to diminish as distance increases beyond the marathon [7] because women have greater fat stores and an improved fat metabolism compared to men and should be more fatigue-resistant than male athletes during ultra distances [41]. In Ultraman Hawaii, running performance during day 3 is the last part of this multi-stage event. During the day, the temperature may increase up to 30°C in Hawaii [42]. Presumably, the athletes might have had only little reserves left for the run after two races of racing. The training and previous experience might provide the athletes with the empirical knowledge to select the optimal pacing strategy with the ability to maintain race pace [21]. During multi-stage races such as Tour the France and Vuelta de Espana [22,23], race intensity is rather high. Rodríguez-Marroyo et al. showed that experienced athletes modulated the intensity of each stage due to the total race duration [23]. Ironman triathletes have a special strategy during the cycling phase dependent upon the wind [43]. In a multi-stage triathlon such as Ultraman Hawaii, a specific pacing strategy is needed including also the aspect of recovery and nutrition between the stages [20,21].

### The age of peak performance

The results of the present study only partly supported the hypothesis that the average age of finishers increased over time. The age of the annual top male finishers increased, while the age of annual top female finishers showed no changes. It must be stated that the increase in the age of the male winner across years was not due to the same athlete. Several different athletes were able to win Ultraman Hawaii [24]. Considering the annual top three finishers, age increased in both women and men. Participation in master athletes in triathlons [44] and ultra-triathlons [5,44,45] increased over the last two decades, so it was not surprising that the mean age increased in finishers. Master athletes were generally defined by the age at which the world record in open elite sport peaks, typically older than 35–40 years [46]. The lack of a comparable increase in the age of female finishers over time might be attributable to the small number of female finishers over 40 years of age or to a low number of female master athletes in general. Overall, the decline in athletic performance with age was greater in women than in men [47]. The age-related decrease in maximal oxygen uptake seemed to be the most consistent contributor to the decline in endurance performance with advancing age, and age-related reduction in lactate threshold might also be important [48]. One reason for the increase in the age of peak performance could be the increasing importance in training and competition strategies [49] as the number of competitors was growing in endurance sport events [2-6,50]. Another reason for the increasing age of peak performance might be the popularity of Ultraman Hawaii, which attracted more athletes and especially more master athletes in recent years.

### Limitations of the present study and practical applications

The major limitation of the present study is the small number of participants (maximum of 40 invited athletes per year) and finishers in the Ultraman Hawaii. This is especially true for female participants who have never made up more than 26% of the total group. The inclusion of individual participants who finished in more than 1 year probably did not strongly influence the results. Another limitation was the lack of information about performance-related factors such as training [34,37], motivation [27], nutrition [20,51,52], fluid metabolism [53,54], previous experience [21,34], weather [55], equipment [35,56], and anthropometry [57,58] of individual participants, which could significantly affect individual performance and overall strength of the athlete. We did not check for disqualification of athletes due to doping. One athlete was disqualified in 1998 due to unsportsmanlike conduct. For practical applications, the fastest finishers improved performance, and the age of the fastest finishers increased in Ultraman Hawaii as has also been found in Ironman Hawaii [59]. However, the fastest finishers in Ironman Hawaii seemed younger than the fastest finishers in Ultraman Hawaii [59]. The upper limits in the age of peak performance in both Ironman Hawaii and Ultraman Hawaii seemed not reached for both women and men.

## Conclusions

The number of female finishers in Ultraman Hawaii increased, while the number of male finishers remained unchanged between 1983 and 2012. Performance by both sexes improved over time, but men remained faster than women. The sex-related difference in overall performance decreased over time from 24.3% to 11.5%. The age of peak performance was higher for men than for women and it increased for both the fastest women and the fastest men. Further investigations are required to better understand the limiting factors of the multi-activities ultra-endurance events taking place over several days.

## Competing interests

The authors declare that they have no competing interests.

## Authors’ contributions

All authors designed the study. The manuscript was written by all authors. All authors read and approved the final manuscript.
